# Computational Approaches for the Discovery of GPER Targeting Compounds

**DOI:** 10.3389/fendo.2020.00517

**Published:** 2020-08-04

**Authors:** Fedora Grande, Maria A. Occhiuzzi, Rosamaria Lappano, Francesca Cirillo, Rita Guzzi, Antonio Garofalo, Yves Jacquot, Marcello Maggiolini, Bruno Rizzuti

**Affiliations:** ^1^Department of Pharmacy, Health and Nutritional Sciences, University of Calabria, Rende, Italy; ^2^Department of Physics, University of Calabria, Rende, Italy; ^3^CNR-NANOTEC, Licryl-UOS Cosenza and CEMIF.Cal, Department of Physics, University of Calabria, Rende, Italy; ^4^Cibles Thérapeutiques et Conception de Médicaments (CiTCoM), CNRS UMR 8038, INSERM U1268, Faculté de Pharmacie de Paris, Université de Paris, Paris, France

**Keywords:** G protein-coupled estrogen receptor 1, estrogen receptors, ligands, drug design, molecular docking, molecular dynamics

## Abstract

Estrogens exert a panel of biological activities mainly through the estrogen receptors α and β, which belong to the nuclear receptor superfamily. Diverse studies have shown that the G protein-coupled estrogen receptor 1 (GPER, previously known as GPR30) also mediates the multifaceted effects of estrogens in numerous pathophysiological events, including neurodegenerative, immune, metabolic, and cardiovascular disorders and the progression of different types of cancer. In particular, GPER is implicated in hormone-sensitive tumors, albeit diverse issues remain to be deeply investigated. As such, this receptor may represent an appealing target for therapeutics in different diseases. The yet unavailable complete GPER crystallographic structure, and its relatively low sequence similarity with the other members of the G protein-coupled receptor (GPCR) family, hamper the possibility to discover compounds able to modulate GPER activity. Consequently, a reliable molecular model of this receptor is required for the design of suitable ligands. To date, convergent approaches involving structure-based drug design and virtual ligand screening have led to the identification of several GPER selective ligands, thus providing important information regarding its mode of action and function. In this survey, we summarize results obtained through computer-aided techniques devoted to the assessment of GPER ligands toward their usefulness in innovative treatments of different diseases.

## Introduction

The multifaceted responses to estrogens are principally mediated by the estrogen receptors (ERs) α and β, which act as transcription factors by binding to estrogen response elements (EREs) located in the promoter regions of target genes (1). Recently, a seven-transmembrane G protein-coupled receptor, known as G protein estrogen receptor (GPER), has attracted the attention of several researcher groups working on the identification of the intricate estrogen routes in different biological systems. A panel of experiences has highlighted the involvement of GPER in various pathophysiological processes. For instance, its role in hormone-dependent cancers has been addressed in several studies, providing a better understanding of the related gene landscape and transduction pathways. In particular, GPER modulates signaling processes leading to the transcription of genes promoting tumor growth *in vitro* and *in vivo*, such as calcium mobilization, cAMP synthesis, the cleavage of matrix metalloproteinases, the transactivation of epidermal growth factor receptor (EGFR) and the activation of PI3K and MAPK transduction pathways (2–11). To date, GPER expression has been correlated with negative cancer features including increased tumor size, distant metastasis and tumor recurrence (12–14). In addition, a bioinformatic analysis of large cohorts of patients has recently demonstrated that GPER expression is correlated with the expression of pro-metastatic genes in ER-negative breast tumors (15). On the basis of the aforementioned findings, this receptor might be considered as a promising therapeutic target for the treatment of diverse types of tumors, including breast cancer. Nevertheless, other studies reached different conclusions (16), therefore indicating that further investigations are required to better appreciate the role exerted by GPER in cancer.

Most estrogens and anti-estrogens are able to bind to GPER and ERs, albeit with a different affinity and even with an opposite action (i.e., agonism vs. antagonism) (10, 17, 18). Considering the interest to identify specific GPER ligands to decipher its unique potential, several successful efforts have been made during the last few years (19–25). In this context, it should be mentioned the intriguing discovery of the indole derivative MIBE, which has the property of binding to and antagonizing the effects of both GPER and ER, thus representing a useful tool toward more comprehensive approaches in estrogen-dependent tumors (22).

**Figure 1:**
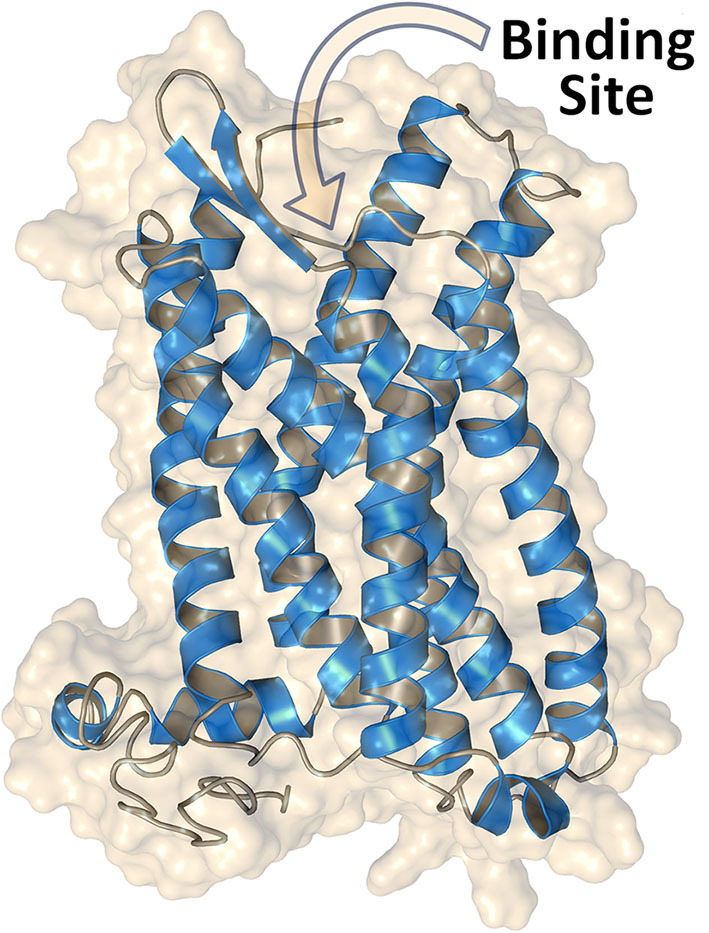
Structure of GPER obtained through homology modeling by using the web server GPCR-I-TASSER (26). The extracellular N-terminal region (residues 1–50) is omitted, and access to the binding site for the ligands is indicated.

The overall structural heterogeneity among agents targeting these receptors constitutes an obstacle to identify agonists or antagonists and to predict their effects. Thus, the design of potent selective GPER ligands and dual ER/GPER inhibitors is still challenging. While the crystallographic structure of the ER ligand-binding domain is available, the detailed structure of GPER remains yet unsolved due to the well-known difficulties in fully characterizing membrane proteins. Nevertheless, a homology model of GPER can be obtained with the help of computational techniques (Figure 1), allowing access to relevant structural information. More importantly, virtual ligand screening approaches and structure-based drug design methods can support experiments aiming to identify new GPER ligands. The results obtained by application of computational techniques are herein summarized toward their adoption as starting point for the design and development of novel active agents.

## The Early Age of Ligand-Based Design for Targeting GPER

**Figure 2:**
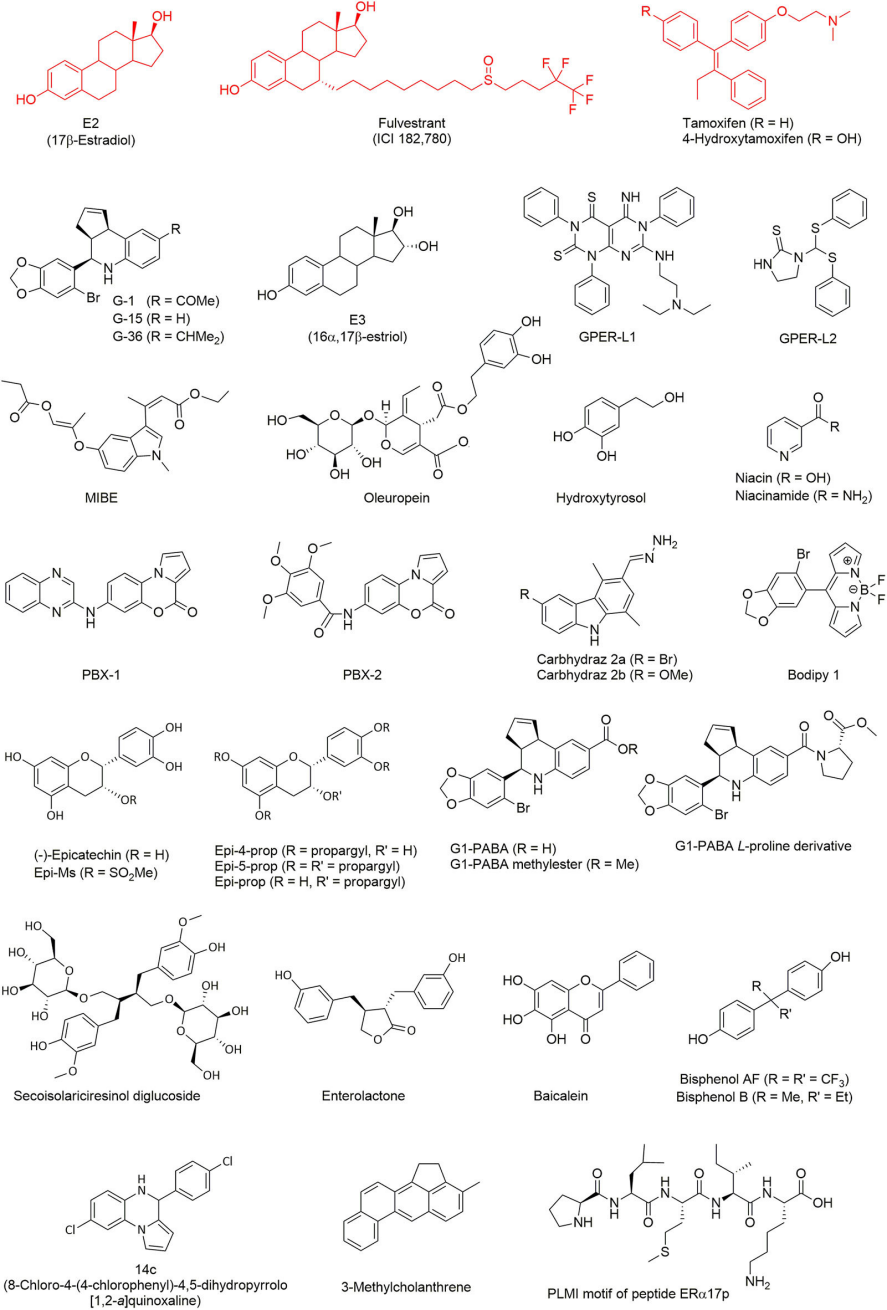
GPER ligands investigated by computational methods. Typical ligands (represented in red), including E2, fulvestrant, and tamoxifen, have been extensively tested in simulation as reference GPER binders. All the other ligands (in black) were discovered or characterized through virtual screening techniques.

With respect to the computational design of GPER ligands, one of the earliest achievements is indisputably the synthesis of the quinoline G-1 (19). To this aim, a library of 10,000 candidate molecules has been analyzed by combining their structural features in common with the archetypal ligand 17β-estradiol (E2). Three distinct categories of criteria were defined: (i) 2D structural patterns, including both symmetric and asymmetric features, (ii) 3D shape analogies and metrics and (iii) pharmacophore-based motifs, including hydrogen-bond donors/acceptors and the possibility of forming hydrophobic interactions. The resulting computational analysis was completed with an *in vitro* biomolecular screening to validate the occurrence of a competitive ligand binding. The molecule G-1 (for the chemical structure of this compound and all the other mentioned throughout the text, see Figure 2) has emerged from this screening funnel as the first GPER-specific agonist able to activate the receptor in cells expressing both GPER and ERs. A similar virtual screening methodology, applied on a larger library with more than 140,000 compounds, led successively to the identification of a high-affinity GPER ligand named G-15, a G-1 analog acting as a selective antagonist (20).

The success of these original ligand-based virtual screenings was facilitated by the limited number of internal degrees of freedom of the studied compounds. These observations referred also to the conformational space of the ligand-binding pocket of the different steroid hormone receptors (27). In the absence of a model for the GPER tertiary structure, the previous works were, therefore, expanded to an “indirect” structure-based approach consisting in the analysis into deeper details of the ERα and ERβ binding sites. In fact, the main concern was to focus on the acetyl moiety of the ER agonist G-1, which is lacking in the antagonist G-15 (21). Molecular docking was then performed to explore the possibility to increase steric clashes within the binding pocket of the ERs through an isopropyl moiety instead of the acetyl group. The resulting compound, named G-36, showed an enhanced selectivity with an antagonist effect toward GPER, and a low-affinity cross-reactivity toward ERα.

## Homology Modeling for Predicting the Structure OF GPER

The possibility of investigating in simple and accurate ways the binding location and the affinity of molecules to GPER is intimately linked with the availability of a correct molecular description of the protein structure. The first crude GPER model was built to predict ligand binding, through computational approaches (28). A more accurate structure of GPER was later used as a support to elucidate a number of “wet-lab” experiments (18, 22, 29). In all cases, a special emphasis was put on providing details about the topology of the putative binding-site of the protein, rather than the whole protein tertiary structure. The 3D structure of GPER was constructed through homology modeling (30, 31), by using the crystal structure of bovine rhodopsin (32) as a template.

Subsequent efforts were devoted on the improvement of the molecular description of the protein structure, with a broader emphasis on the description of the overall protein architecture. Consistent results were achieved by using as a template the X-ray structure of the β_2_-adrenergic receptor, which was found to possess a higher degree of homology with GPER, when compared to bovine rhodopsin. The crystal structures of both the active and inactive states of the β_2_-adrenergic receptor were available (33, 34), allowing to model different conformations of GPER in complex with agonists or antagonists (35).

Alternatively, GPER has been modeled (36) by using as a template the crystallographic structure of the chemokine receptor CXCR4 (37). It was also possible to identify some structural differences between agonists and antagonists, depending on their ability to form hydrogen bonds with key residues located in specific transmembrane helices. The quality level of this new GPER model was further improved through molecular dynamics (MD) simulations (36, 38, 39), which were helpful to take into account the internal dynamics of the protein, concomitantly to the contribution of the surrounding lipid matrix.

A more comprehensive view of the 3D structure of GPER has been recently obtained by using the web server GPCR-I-TASSER (26, 40), which is a computational suite derived from the parent I-TASSER package (41), one of the most popular on-line resources for protein structure prediction and refinement. A major strength of the algorithm used in GPCR-I-TASSER is that it was specifically devised for the prediction of GPCRs and, as such, it works with a dedicated knowledge-based force field as a guide for an accurate assembly of the receptor structure. A number of works (25, 38, 42, 43) have adopted this computational tool to design high-quality GPER models (see again Figure 1). High-quality and tailored software, together with the growing number of experimentally-determined GPCR structures that can be used as GPER templates, are contributing to make homology modeling algorithms more accurate in predicting the receptor structure.

## The Beginning of Structure-Based Design of Ligands for GPER

In the last decade, one of the major aims of our research group has been the identification of novel GPER ligands supported by computational drug discovery approaches. A first result (18) was the identification of 16α,17β-estriol (E3) as a GPER antagonist (see Figure 2). This compound, which corresponds to a final metabolite of E2 (44), is one of the three natural estrogens produced by the human body (i.e., estradiol, estrone, and estriol). It is of note that E3 can also be biosynthesized from estrone sulfate as well as from testosterone and androstenedione (44).

A computational analysis also allowed the discovery of two new molecules (22), named GPER-L1 and GPER-L2, both acting on GPER as selective agonists but unable to bind and activate ERs. These two ligands were selected through a typical process of virtual screening starting from a chemical library including more than 300 molecules. Despite strong differences in their chemical structure, they share some crucial features such as the ability of exposing a phenyl ring into a hydrophobic protein pocket and, thereby, of forming stabilizing π-π stacking interactions. Due to their selectivity, these molecules contribute to increase our understanding of the function of various cancer phenotypes, through different estrogen-targeted receptors.

In sharp contrast, MIBE was identified as a unique case of dual antagonist for both GPER and ERα (29), by using molecular docking. The possibility of targeting simultaneously GPER and ERα is particularly interesting from a pharmacological point of view, because active compounds antagonizing both proteins could be useful in tackling breast carcinomas at the initial stage or during their progression. In a subsequent work (45), it was demonstrated that oleuropein and hydroxytyrosol, two natural phenols, were able to bind GPER. Flexible molecular docking calculations were performed with these two molecules, considering free rotation of seven bulky side chains in the binding site of the receptor, which was especially required to accommodate the relatively large molecular structure of oleuropein. The following experiments demonstrated that both ligands act as GPER inverse agonists in ER-negative and GPER-positive SkBr3 breast cancer cells, as recently found with the peptide ERα17p (25).

A virtual screening campaign on a library of chemical fragments demonstrated the binding of niacin (also known as nicotinic acid, vitamin B3, or vitamin PP) and of its amide form niacinamide (or nicotinamide) to GPER and niacin receptor GPR109A/HCA2 (46). The latter is a subtype of the receptor GPR109 that mainly, although not exclusively, mediates the action of niacin (but not of niacinamide). Sequence alignment revealed a lack of homology between GPER and GPR109A, at least in their ligand-binding sites, thus the anchoring of these two molecules to both GPER and GPR109A was not obvious. Nevertheless, molecular docking suggested some similarities in the binding mode of the pyridine ring of both compounds. The presence of two important arginine residues able to form hydrogen bonds with the carboxylic acid moiety of niacin, allowing GPR109A specificity, were also highlighted. Both niacin and niacinamide were demonstrated to exert an agonist activity toward GPER in the successive experiments.

Computational techniques further helped to design two novel benzopyrroloxazines (24) acting as selective GPER antagonists. The starting point was the virtual screening of a compound library and the identification of a rigid molecular structure closely resembling the chemical skeleton of other known GPER ligands. This structure was used as a template and two derivatives, named PBX1 and PBX2, were demonstrated to bind to GPER. To this aim, flexible molecular docking was performed by allowing the rotation of the dihedral angles in the side chain of eight residues within the protein-binding site.

In a separate study (23), two novel carbazole derivatives were synthesized. While both of them did not activate the classical ERα, one of them, abbreviated as carbhydraz 2a, showed a favorable affinity for GPER, as shown by docking assays with seven flexible residues. This compound was then shown to display an agonistic response on GPER.

As a final example, a rational design has been completed with the first GPER selective fluorescent organoboron probe (47), which consists in a boron-dipyrromethene difluoride derivative. In this case, the starting molecular template was a bromobenzodioxolyl substituent, which is also present in the structure of G-1 and constitutes a key motif for GPER binding. Using molecular docking, the obtained compound, named Bodipy 1, was predicted to share a binding mode similar to G-1, by interacting with the key protein residue Phe-208 and by forming π-π stacking with its bromobenzodioxole moiety. Bodipy 1 was later demonstrated to compete with niacin in ER-negative and GPER-positive SkBr3 breast cancer cells.

## The Current Area OF Computational Methods for Studying GPER

The study of GPER through theoretical modeling approaches has lately benefited from a number of improvements, including the use of targeted simulations to capture important aspects of the protein dynamics. Molecular docking on GPER structures extracted from all-atom MD has demonstrated (48) that the natural polyphenol (–)-epicatechin (see Figure 2) has the ability to anchor to this receptor with a binding mode similar to the agonist G-1. It is interesting to note that flavonoids sharing structural similarities to estrogens, such as genistein and other phytoestrogens (49–51), not only bind but also activate the classical receptors ERα and ERβ. In contrast, and in spite of its evident structural analogies to these phytochemicals, (–)-epicatechin fails to bind ERα and ERβ. Since (–)-epicatechin can associate within the GPER binding pocket, an important role of this receptor on cardiovascular system protection seems likely. In a subsequent study (52), (–)-epicatechin derivatives were obtained where the phenol and alcohol groups are functionalized with a propargyl or a mesyl group. The resulting compounds, i.e., Epi-4-prop, Epi-5-prop, Epi-prop and Epi-Ms, were investigated by docking methods on the GPER structures obtained through MD. Strikingly, it was observed that the alkyne function of the propargyl was prone to generate additional interactions in the receptor-binding site and to enhance the GPER agonistic activity, when compared to the parent compound. Later, MD simulations showed that Epi-4-prop and Epi-5-prop share with (–)-epicatechin similar interactions at the GPER binding site (53).

A number of additional GPER binders have also been identified using theoretical modeling calculations. It is the case of G1-PABA (54), a compound part of a small series of G1 analogs, in which the acetyl moiety is replaced with a carboxyl group. The same pharmacophore core was further used to obtain a G1-PABA methylester and *L*-proline derivative, with similar structural and energetic binding properties (55). All these newly synthesized compounds were validated *in vitro* in experimental assays using breast cancer cell lines. Another example is secoisolariciresinol diglucoside, a phytoestrogen extracted from flaxseed and able to suppress benign prostatic hyperplasia (56), which was indirectly investigated through molecular docking of its mammalian metabolite enterolactone (57). Similarly, the binding mode to both GPER and ERα of baicalein, a flavonoid derived from the roots of a medicinal herb, has been extensively studied (58). Molecular docking analysis was especially focused on the hydrogen-bond network favoring the ligand binding. The observation that GPER appeared to mediate the estrogenic effects of some bisphenol A analogs was the starting point of another computational study (59), which predicted a favorable binding of bisphenol AF and bisphenol B. Experimental assays have confirmed the agonistic activity of these compounds, supporting the hypothesis of their disrupting action on the GPER-mediated pathway. These observations clearly highlight the lack of selectivity of phytoestrogens (60).

Similarly to G-15, theoretical methods have also been used to identify new compounds (61) with a selective anti-proliferative activity against GPER-expressing breast cancer cells. A virtual screening campaign was carried out on a chemical library of about 1,000 compounds, in search of molecules showing a binding mode close to G-15 and G-36. They were selected on the basis of their binding score and by visual inspection, as well as their ability to form polar contacts with previously identified GPER residues. Four different chemical scaffolds were found. A particularly promising compound, named 14c and based on one of these scaffolds, has been proposed as a starting point for a future hit-to-lead optimization process.

By using combined docking and MD simulations approach, the association of the chemical carcinogen 3-methylcholanthrene with GPER has been recently studied (43). This environmental pollutant, which is generated by incomplete combustion processes including cigarette smoke, was known to bind to both ERα and the aryl hydrocarbon receptor (AhR), stimulating thereby a functional interaction between these two receptors. The results pointed out to a functional crosstalk and a cross-stimulation between GPER and AhR. Computational methods have also been used to investigate the binding to GPER of the peptide ERα17p, which encompasses a part of the hinge region/AF2 domain of the human ERα (25, 62). This peptide acts as a GPER inverse agonist and shows anti-proliferative effects in breast cancer cells and a decrease in the volume of breast tumors in xenografted mice (63). The N-terminal PLMI motif of this peptide presents some chemical analogies with the GPER antagonist PBX1 and exerts the same anti-proliferative potency as the whole length peptide (25, 62), suggesting strongly that this region corresponds to the active motif. Due to the large number of rotatable bonds in ERα17p, MD simulations were necessary to map the conformational landscape of the receptor-peptide molecular system. The fact that a specific amino acid drives the anchoring of ERα17p opens the way to the possibility of modulating GPER by using peptide-based compounds. It is particularly notable that ERα17p is, to the best of our knowledge, the first peptidic GPER modulator.

## Conclusions

GPER is increasingly recognized as a mediator of different estrogen-dependent pathophysiological responses, such as those that characterize cancer progression. The persistent difficulty in obtaining an experimental structure of the native structure of this membrane receptor, let alone in complex with any endogenous or exogenous ligands, has prompted an abundance of theoretical studies to clarify its conformation and binding properties. In this context, molecular modeling of GPER ligands has demonstrated that targeting this receptor with computational methods is feasible. Accordingly, a number of compounds has been defined toward the development of innovative molecular modulators of GPER action in different biological systems. In particular, the first identified GPER agonist, G-1, is currently undergoing phase 1 clinical trials for its immunomodulatory and antineoplastic properties. In this respect, the findings recapitulated and discussed herein could be useful in order to clarify the potential role of GPER in cancer and other diseases, and the advantages of computational approaches to drive drug discovery for this target.

## Author Contributions

All the authors have contributed to prepare the manuscript and approved it for publication.

## Conflict of Interest

The authors declare that the research was conducted in the absence of any commercial or financial relationships that could be construed as a potential conflict of interest.

## Abbreviations

AhR: aryl hydrocarbon receptor
E2: 17β-estradiol
E3: 16α 17β-estriol
EGFR: epidermal growth factor receptor
ER: estrogen receptor
ERE: estrogen response element
GPCR: G protein-coupled receptor
GPER (or GPR30): G protein-coupled estrogen receptor 1
MD: molecular dynamics.
